# Time-dependent Diffusion MRI for Predicting Response to Induction Chemotherapy in Nasopharyngeal Carcinoma

**DOI:** 10.1148/rycan.250579

**Published:** 2026-05-08

**Authors:** Huanhuan Ren, Diwei Shi, Xiaoxia Wang, Qian Xu, Yao Huang, Haojie Song, Junhao Huang, Xinying Ren, Xiaosong Lan, Yong Tan, Hong Yu, Lisha Nie, Daihong Liu, Jiuquan Zhang

**Affiliations:** ^1^Department of Radiology, Chongqing University Cancer Hospital, No. 181, Hanyu Rd, Shapingba District, Chongqing 400030, China; ^2^Research Institute of Tsinghua University in Shenzhen, Shenzhen, China; ^3^Tsinghua Shenzhen International Graduate School, Tsinghua University, Shenzhen, China; ^4^Department of Biomedical Engineering, School of Medicine, Shenzhen University, Shenzhen, China; ^5^GE HealthCare MR Research, Beijing, China

**Keywords:** Nasopharyngeal Carcinoma, Response, MRI, Induction Chemotherapy

## Abstract

**Purpose:**

To validate the reliability of time-dependent diffusion MRI (td-dMRI)–derived indicators in nasopharyngeal carcinoma (NPC) and determine whether microstructural parameters derived from td-dMRI can help noninvasively predict response to induction chemotherapy.

**Materials and Methods:**

In this prospective study, participants with locally advanced NPC underwent pretreatment td-dMRI between December 2023 and February 2025. The data were randomly stratified and divided into a training set and an internal test set at a 7:3 ratio. According to Response Evaluation Criteria in Solid Tumors 1.1, all participants were classified as responders or nonresponders. Four imaging microstructural parameters with the limited spectrally edited diffusion-derived microstructural parameters (cell diameter *d*, cellularity, intracellular volume fraction *f*_in_, and extracellular diffusivity *D*_ex_) and three apparent diffusion coefficient (ADC) values (the ADC with the pulsed gradient spin-echo sequence, the ADC at 20 Hz, and the ADC at 40 Hz) were calculated. Repeatability was evaluated. MRI-derived parameters were compared with pathologic microstructural metrics. In the training set, univariable and multivariable logistic regression analyses were performed to identify parameters associated with induction chemotherapy response; predictive models were constructed.

**Results:**

In total, 220 participants with NPC were enrolled (median age, 53.0 years [IQR, 48.8–58.0 years]; 155 male participants), including 154 in the training set and 66 in the internal test set. Participants were divided into responders (*n* = 151) and nonresponders (*n* = 69). The repeatability study revealed within-subject coefficient of variation values of 4.56%–10.15% and repeatability coefficient values of 0.12–1.71. Moreover, the td-dMRI–derived parameters correlated well with pathologic measurements (*r* = 0.27–0.67; *P* < .05). In the training set, univariable and multivariable logistic regression analyses revealed that the tumor-stroma ratio (TSR) and td-dMRI–derived cellularity were independent predictors. The combined model integrating the TSR and cellularity achieved an AUC of 0.82 (95% CI: 0.75, 0.89) in the training set and 0.76 (95% CI: 0.63, 0.87) in the internal test set.

**Conclusion:**

td-dMRI–derived microstructural parameters, particularly TSR and tumor cellularity, showed good performance in predicting response to induction therapy in individuals with NPC.

**Keywords:** Nasopharyngeal Carcinoma, Response, MRI, Induction Chemotherapy

*Supplemental material is available for this article.*

© The Author(s) 2026. Published by the Radiological Society of North America under a CC BY 4.0 license.

## Graphical abstract

**Figure:**
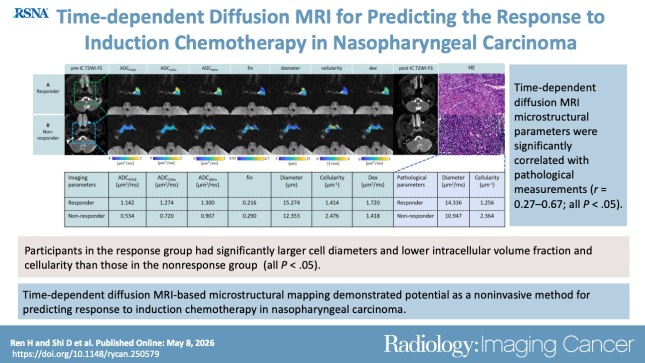

**Summary**

Time-dependent diffusion MRI–based microstructural mapping demonstrated potential as a noninvasive method for predicting response to induction chemotherapy in nasopharyngeal carcinoma.

**Key Points**

■ In this prospective observational study of 220 participants with nasopharyngeal cancer, time-dependent diffusion MRI (td-dMRI) parameters demonstrated good repeatability (within-subject coefficient of variation: 4.56%–10.15%; repeatability coefficient: 0.12–1.71).
■ td-dMRI microstructural parameters were correlated with pathologic measurements (*r* = 0.27–0.67; all *P* < .05).
■ A combined model incorporating the tumor-stroma ratio and cellularity achieved an area under the receiver operating characteristic curve of 0.82 in the training set and 0.76 in the internal test set for predicting response to induction chemotherapy.

## Introduction

The primary treatment approach for locally advanced nasopharyngeal carcinoma (NPC) is induction chemotherapy followed by concurrent chemoradiotherapy (1). A randomized controlled trial demonstrated that induction chemotherapy responders enjoyed a significant survival advantage, with an absolute 5-year overall survival improvement of 27%–38.5% over nonresponders (2). More generally, chemotherapy resistance is typically linked to poor prognosis (3). However, only 67.6%–94.5% of patients with NPC exhibit an optimal response to induction chemotherapy (4). Uncertainty in treatment response may lead to either unnecessary or delayed interventions. Therefore, there is an urgent need for reliable methods to accurately identify chemotherapy-sensitive patients with NPC before treatment, as such methods would hold considerable promise for improving clinical management.

MRI is crucial for evaluating treatment response in NPC. Several studies have assessed the value of the pretreatment diffusion weighted imaging–derived apparent diffusion coefficient (ADC) for predicting chemotherapy responses in NPC, but their findings have been inconsistent (5–7). This may be because the ADC is typically influenced by multiple factors, including cell size and intrinsic diffusivity (8), which represents the average diffusion arising from different compartments.

The emerging time-dependent diffusion MRI (td-dMRI) technique has enabled the quantification of microstructural properties such as cell diameter, cellularity, and the intracellular fraction (*f*_in_), which conventional diffusion-weighted imaging cannot objectively capture (9). Assessing measurement precision is a crucial but usually overlooked step in biomarker translation (10,11). Repeatability is essential for diagnostic and prognostic utility (12). Although the repeatability of td-dMRI has been demonstrated in the rat liver (13), to our knowledge, no existing studies have evaluated the repeatability of td-dMRI in the human nasopharynx. Because the complex anatomy and susceptibility differences of the nasopharynx cause distortions at diffusion-weighted imaging (13), a repeatability study is necessary to ensure clinical reliability.

Cell size as measured with td-dMRI reflects tumor risk stratification and treatment response, and cellularity reflects the proliferative activity of the tumor (14). The feasibility of these key indicators for distinguishing tumor histologic grades and predicting treatment response has been demonstrated in breast cancer (15), prostate cancer (8), and glioma (14). Therefore, we hypothesized that these parameters could also provide valuable insights at the microscopic level for predicting chemotherapy response in NPC.

Accordingly, the aims of our study were to validate the reliability of td-dMRI–derived indicators in NPC and to investigate the feasibility of using microstructural parameters derived from td-dMRI sequences to predict the induction chemotherapy response in individuals with NPC.

## Materials and Methods

### Study Participants

This prospective study was approved by the local ethics committee and conducted in compliance with the Declaration of Helsinki. In addition, all participants provided written informed consent to undergo td-dMRI measurements alongside routine MRI examinations.

Consecutive participants with NPC were prospectively recruited between December 2023 and February 2025. The inclusion criteria were as follows: *(a)* histopathologically confirmed NPC, *(b) *stage III–IVa disease according to the eighth edition of the American Joint Committee on Cancer (AJCC) staging system, *(c)* complete induction chemotherapy and neck and nasopharyngeal MRI before and after induction chemotherapy, and *(d)* no other primary tumors. The exclusion criteria were as follows: *(a)* unmeasurable (≤5 mm) NPC on pretreatment T1-weighted images, *(b)* poor image quality insufficient for analysis (16), and *(c)* incomplete clinical data.

On the basis of published criteria, the participants were classified into high- and low-expression (≥50% vs <50%) groups according to the Ki-67 value and the tumor-stroma ratio (TSR) (17–20). The TSR was assessed on representative hematoxylin-eosin–stained sections containing both tumor and stromal components. First, the entire tumor section was scanned at low magnification to identify the area with the highest stromal content. TSR was subsequently evaluated at higher magnification within a 3-mm^2^ field, ensuring that the stroma was entirely surrounded by tumor cells. The methodologic specifications for TSR assessment are detailed in Appendix S1.

### MRI Acquisition

All scans were obtained with a 3.0-T MRI scanner (Premier; GE HealthCare) with a maximum gradient strength of 80 mT/m and a maximum slew rate of 200 mT/m/msec, using a dedicated 32-channel head-neck coil. td-dMRI acquisitions included two sets of vendor-developed oscillating gradient spin-echo sequences (21) at 20 Hz and 40 Hz and one pulsed gradient spin-echo (PGSE) sequence. All three sequences were implemented with AIR Recon DL (GE HealthCare) deep learning reconstruction embedded for k-space–based denoising (22). The entire td-dMRI scan lasted approximately 7.5 minutes. The other acquisition sequences included T1-weighted, T2-weighted, T2-weighted with fat suppression, and contrast-enhanced T1-weighted imaging. For details of the imaging protocols, please refer to Tables S1 and S2.

The participants recruited from December 2023 to February 2024 underwent two td-dMRI examinations on the same day (test-retest study), with identical positioning and identical scanning parameters. The interval between the two acquisitions ranged from 0.57 to 0.7 hours. According to published studies, at least 35 test-retest subjects are necessary for providing 95% coverage of the true biomarker value (23,24).

### Model Construction

All participants were stratified and randomly divided into a training set and an internal test set at a 7:3 ratio. Univariate logistic regression analysis was first performed to identify variables associated with response to induction chemotherapy (*P* < .05). Multivariable logistic regression analysis was then conducted to determine independent predictors of the response to induction chemotherapy. In the multivariable analysis, male sex was used as the reference category for the sex variable, and the smallest category was used as the reference for all other categorical variables. Based on the above independent predictors, single-parameter models and multiparameter combined models were constructed using logistic regression to predict the response to induction chemotherapy. The code used for random stratified sampling and model construction in this study is available at *https://github.com/YaoHuang1123/NPC-Statistical-Model-Training-and-Evaluation*.

### Image Analysis and Assessment

**Qualitative assessment of image quality**

The quality of the td-dMRI images was independently evaluated by two radiologists (X.R. and D.L., with 4 and 5 years of experience in head and neck MRI, respectively). The two radiologists, both blinded to the participants’ clinical data, individually evaluated the image quality using a five-point scoring system (25). The image quality was scored as follows: 1, poor; 2, fair; 3, moderate; 4, good; and 5, excellent (26).

For assessment of intraobserver agreement, the same observer independently evaluated image quality on two separate occasions, with a 4-week interval between assessments. Interobserver agreement in assessing image quality for the three td-dMRI sequences was evaluated using kappa (κ) analysis. The level of agreement was categorized as follows: poor (κ < 0.21), fair (κ = 0.21–0.40), moderate (κ = 0.41–0.60), substantial (κ = 0.61–0.80), or excellent (κ = 0.81–1.00) (27).

The two radiologists independently delineated the volumes of interest (VOIs) on images with an image quality score of 3 or higher, while being fully blinded to patients’ clinical information and pathologic results. Interreader agreement of VOI delineation was quantitatively assessed using the Dice similarity coefficient.

The Dice coefficient was defined as twice the volume of the intersection of the two VOIs divided by the sum of the volumes of the VOIs delineated by the two radiologists. Dice coefficient values range from 0 to 1, with values closer to 1 indicating higher agreement. A Dice coefficient greater than 0.70 was considered indicative of good interreader agreement.

**Calculation of Microstructural Parameters Derived from td-dMRI**

A radiologist (J.H., with 13 years of experience) who was blinded to all participants’ data manually delineated the VOIs of nasopharyngeal tumors on the basis of the 40-Hz oscillating gradient spin-echo images obtained at a *b* value of 0 sec/mm^2^ while deliberately excluding necrotic tissue; the boundary voxels were also excluded to avoid any risk of partial volume effects (28).

All diffusion-weighted signals were normalized by dividing them by the unweighted signals. The normalized signals were fitted with the imaging microstructural parameters using limited spectrally edited diffusion (Appendix S2) approach to calculate quantitative parameters, including cell diameter *d*, cellularity, intracellular volume fraction *f*_in_, and extracellular diffusivity *D*_ex_. The ADC values of three different sequences—the PGSE sequence (hereafter, ADC_PGSE_), the 20-Hz sequence (hereafter, ADC_20Hz_), and the 40-Hz sequence (hereafter, ADC_40Hz_) (29)—were obtained with the monoexponential model. Furthermore, an additional ADC-related metric, the relative ADC change, was calculated as follows: (ADC_40Hz−_ − ADC_PGSE_)/ADC_PGSE_ × 100%; this parameter was included given its potential clinical efficacy in published studies. The intracellular diffusivity was set as 1.56 µm^2^/msec, while the other parameter constraints adhered to physiologically plausible ranges (21). All of the above imaging metrics were obtained within each voxel and then averaged over the whole VOI.

**Repeatability of Quantitative Parameters Derived from td-dMRI**

The reliability of all microstructural parameters and ADC-related metrics was evaluated using intraclass correlation coefficients (27). Two radiologists (H.R. and J.H., with 8 and 13 years of experience, respectively), both of whom specialized in head and neck tumors and were blinded to the study design and diagnoses, independently performed the measurements for all participants. To assess intraobserver reliability, the senior radiologist repeated the measurements after 4 weeks. Additionally, on the basis of the VOIs delineated by the two radiologists, we calculated the interobserver variability for VOI placement.

Bland-Altman analysis was used by author (H.Y.) to further evaluate the repeatability of all the metrics from the td-dMRI in the test-retest study (which included 54 participants) (30,31).

**Histopathologic Analysis of Hematoxylin-Eosin–stained Slides**

The hematoxylin-eosin–stained whole-slide images were reviewed by a pathologist with 11 years of experience who was blinded to the MRI findings. After pretraining (Appendix S4), a generative adversarial network was used for automated nuclear segmentation of each slide (32). Quantitative histologic metrics, including *f*_in_, cell diameter, and cellularity, were calculated using previously described methods (15). The Pearson correlation coefficients between MRI-derived and histology-based microstructural parameters were calculated.

**Tumor Response**

For details regarding the induction chemotherapy regimens of all participants, please refer to Appendix S3. The tumor response was assessed on MR images at baseline and after two or three cycles of induction chemotherapy. One senior radiologist reviewed and confirmed the response assessments. Postinduction chemotherapy response was categorized into complete response, partial response, stable disease, and progressive disease according to the Response Evaluation Criteria in Solid Tumors (version 1.1) (Appendix S3). Participants with complete response and partial response were categorized as responders, while those with stable disease and progressive disease were categorized as nonresponders.

**Statistical Analysis**

The minimum required sample size was 59, calculated using PASS 15 software by a statistician (H.Y., with 7 years of experience in statistics) based on a null hypothesis of area under the receiver operating characteristic curve (AUC) of 0.50, an alternative hypothesis of an AUC of 0.75, a responder proportion of 70%, a nonresponder proportion of 30%, and a statistical power of 90%.

Group comparisons were implemented with the independent *t* test (reported as means ± SDs) or the Mann-Whitney *U* test (reported as medians and IQRs) for continuous variables. Categorical variables (reported as numbers of participants and percentages) were compared using the χ^2^ test or the Fisher exact test.

In the training set, univariable analysis was performed to screen features associated with response to induction chemotherapy, and odds ratios (ORs) were calculated for each variable. Variables with a *P* value less than .05 were included in the multivariable analysis to identify independent predictors. The optimal cutoff value was determined using the Youden index. The AUC, specificity, and sensitivity were used to evaluate the predictive efficacy.

After model construction and comparison of predictive performance across models, the model with the best performance was selected for subgroup analyses based on the principle of optimal performance. Subgroup analyses were stratified according to key clinical characteristics, including sex, AJCC stage, and Epstein-Barr virus (EBV) DNA copy number, to evaluate the consistency and robustness of the model across different clinical subgroups.

The 95% CI was calculated with the bootstrap method (1000 intervals). The significance threshold was set at a two-tailed *P* < .05. Statistical analyses were performed by an author (H.Y.) using GraphPad Prism (version 9.5), Python (version 3.7.1), and R (version 4.3.1; R Foundation for Statistical Computing).

## Results

### Participant Characteristics

**Figure 1:**
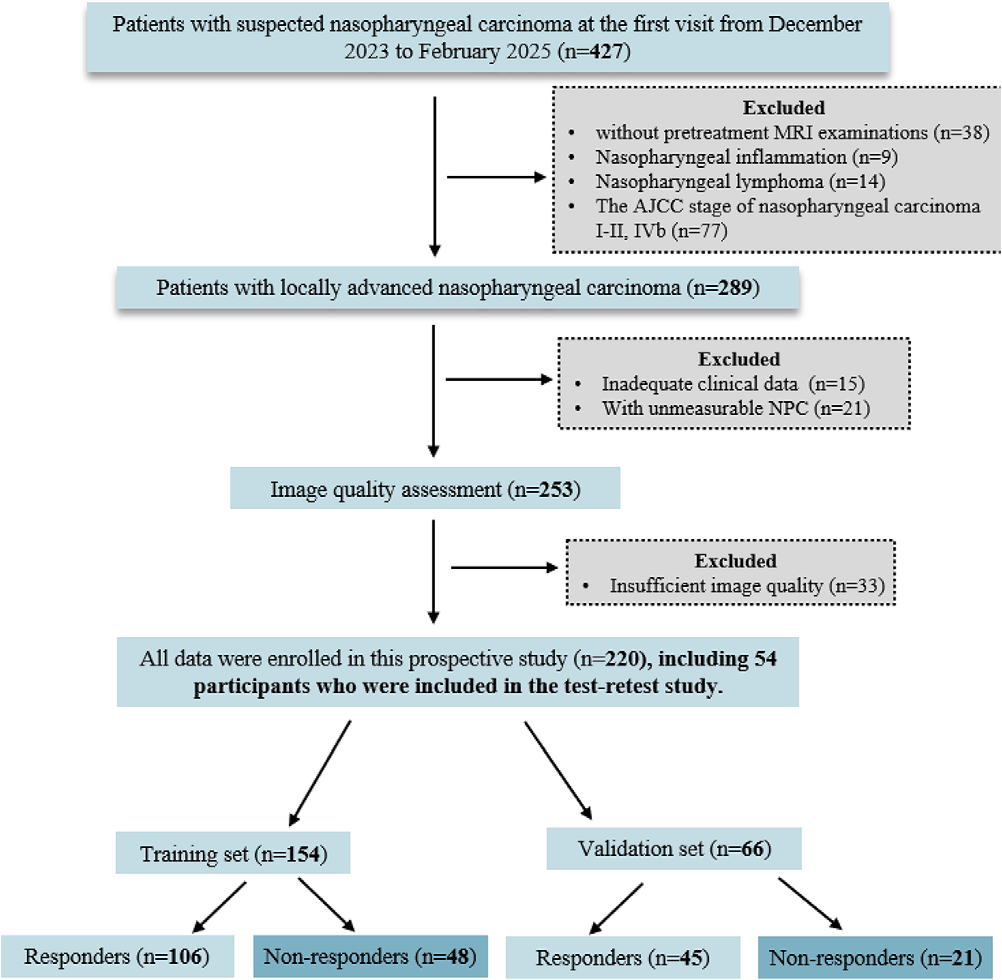
Flowchart shows participant enrollment. AJCC = American Joint Committee on Cancer, NPC = nasopharyngeal carcinoma.

The study included 220 participants (median age, 53.0 years [IQR, 48.8–58.0 years]; 155 male and 65 female participants). A flowchart of the participant selection process is shown in Figure 1. Between December 2023 and February 2025, a total of 427 patients with suspected NPC were initially identified. First, 138 patients were excluded, including 38 without pretreatment MRI examinations, nine with nasopharyngeal inflammation, 14 with nasopharyngeal lymphoma, and 77 with non–locally advanced NPC. An additional 36 patients were subsequently excluded due to incomplete clinical data (*n* = 15) or nonmeasurable lesions (*n* = 21). Of the remaining 253 patients, 33 were further excluded because of poor image quality. Ultimately, 220 patients were included in the final analysis. The clinical and pathologic characteristics of the participants in the training and internal test sets are presented in Table 1. No evidence of differences was observed between the two groups for any of the variables (all *P* > .05).

**Table 1:** Participant Characteristics in the Training Set

Characteristic	Responders (*n* = 106)	Nonresponders (*n* = 48)	*P* Value
Age (y)	53.50 (49.0–58.0)	54.0 (48.5–60.5)	.35*
Male sex	70 (66.0)	36 (75.0)	.27^†^
Albumin level (g/L)	46.7(40.1–52.3)	48.5(42.4–55.6)	.23*
T stage			.07^†^
T1	8 (7.5)	2 (4.2)	
T2	22 (20.8)	5 (10.4)	
T3	31 (29.2)	24 (50.0)	
T4	45 (42.5)	17 (35.4)	
N stage			.32^†^
N0	6 (5.7)	2 (4.2)	
N1	44 (41.5)	13 (27.1)	
N2	35 (33.0)	20 (41.7)	
N3	21 (19.8)	13 (27.1)	
AJCC stage			.95^†^
III	48 (45.3)	22 (45.8)	
IVa	58 (54.7)	26 (54.2)	
EBV DNA (copies/mL)			.06^†^
<4000	74 (69.8)	26 (54.2)	
≥4000	32 (30.2)	22 (45.8)	
Tumor volume (cm^3^)	9.44 (6.25–14.68)	9.94 (6.33–15.15)	.27*
Ki-67 index			0.34^†^
≥50%	40 (37.7)	16 (33.3)	
<50%	66 (62.3)	32 (66.7)	
TSR			.02^†^
≥50%	41 (38.7)	28 (58.3)	
<50%	65 (61.3)	20 (41.7)	
Chemotherapy cycle			.26^†^
Two cycles	71 (67.0)	35 (72.9)	
Three cycles	35 (33.0)	13 (27.1)	

Note.—For categorical variables, data are numbers of participants, with percentages in parentheses. For continuous variables, data are medians, with IQRs in parentheses. T stage and N stage were determined according to the eighth edition of the American Joint Committee on Cancer (AJCC) staging system for head and neck cancer. EBV DNA = copy number of Epstein-Barr virus DNA, TSR = tumor-stroma ratio.

* Determined with the Mann-Whitney *U* test or *t* test.

^†^ Determined with the χ^2^ test or Fisher exact test.

### Image Quality Assessment

**Reader agreement for the qualitative assessment of image quality**

Among the 289 participants with locally advanced NPC, 15 individuals had incomplete clinical data and 21 individuals had unmeasurable NPC lesions (Fig 1). The remaining participants (*n* = 253) underwent an evaluation of td-dMRI image quality. The image quality score was less than 3 for 33 participants, whereas the images of the other 220 participants were suitable for further analysis. The reader agreement for the qualitative image quality assessment is shown in Figure S1 and Table S3. Both intra- and interreader agreement were substantial (0.98 ≤ κ ≤ 0.99 and 0.97 ≤ κ ≤ 0.99, respectively; both *P* < .001). Representative images from the three td-dMRI sequences in a participant with NPC are shown in Figure S2.

**Reader agreement of quantitative measurements**

The intra- and interreader agreement results (intraclass correlation coefficients) for all microstructural parameters and ADC values are summarized in Table S4. The intrareader agreement coefficients exceeded 0.88, while the interreader agreement coefficients were all above 0.74 (all *P* < .001). The mean Dice coefficient for VOI placement between the two radiologists was 0.821 ± 0.054.

**Repeatability assessment of the test-retest study**

The limits of agreement from the test-retest study are summarized in Table 2 and Figure S3, which show that the data points in each plot are generally distributed around the mean difference line, with only a few falling outside the –1.96 to +1.96 SD range for all microstructural parameters and ADC values. Notably, none of the repeatability coefficients exceeded 1.71, and none of the within-subject coefficients of variation exceeded 10.15%.

**Table 2:** Reproducibility Analysis of Microstructural Parameters and ADC Values from Time-Dependent Diffusion MRI for Radiologist 1 in the Test-Retest Study

Parameter	Mean Difference	95% Bland-Altman Limit		Repeatability Coefficient	wCV (%)*
		Lower	Upper		
*f*_in_	0.01	−0.11	0.12	0.12 (0.10, 0.14)	8.40
Diameter	−0.02	−1.74	1.71	1.71 (1.42, 2.14)	4.56
*D*_ex_	0.01	−0.56	0.58	0.57 (0.47, 0.71)	10.15
Cellularity	0.08	−0.89	1.04	0.97 (0.80, 1.21)	9.15
ADC_PGSE_	−0.02	−0.28	0.24	0.26 (0.22, 0.33)	10.13
ADC_20Hz_	−0.01	−0.27	0.25	0.26 (0.22, 0.33)	9.13
ADC_40Hz_	−0.01	−0.22	0.21	0.21 (0.18, 0.27)	6.49
rADC	0.03	−0.23	0.28	0.25 (0.21, 0.32)	9.36

Note.—Numbers in parentheses are 95% CIs. ADC = apparent diffusion coefficient, ADC_PGSE_ = ADC value with the pulsed gradient spin-echo sequence, ADC_20Hz_ = ADC value at 20 Hz, ADC_40Hz_ = ADC value at 40 Hz, *D*_ex_ = extracellular diffusivity, *f*_in_ = intracellular fraction, rADC = relative ADC change, wCV = within-subject coefficient of variation.

* Within-subject SD method.

### Validation of td-dMRI–based Microstructural Parameters against Histopathologic Microstructural Parameters

**Figure 2:**
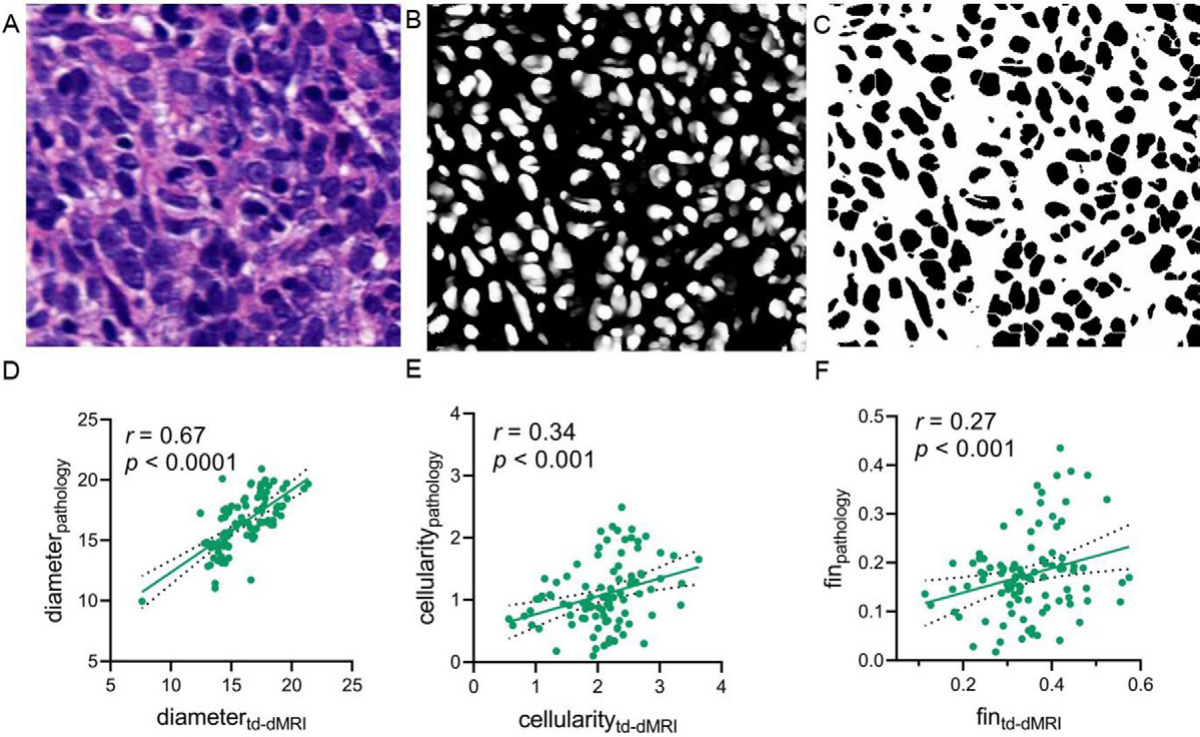
Correlation between pathology-based microstructural properties (*n* = 97) and time-dependent diffusion MRI (td-dMRI)–derived microstructural parameters. **(A)** Photomicrograph (hematoxylin-eosin stain; original magnification, ×40) shows a pathologic specimen from one participant. **(B)** Photomicrograph (hematoxylin-eosin stain; magnification, ×40) shows nuclei that were segmented by a generative adversarial network. **(C)** The pathologic microstructural properties were automatically quantified from the same image. **(D–F)** Graphs show the correlations between the td-dMRI–derived **(D)** diameter, **(E)** cellularity, and **(F)** intracellular fraction (fin) and the pathologic examination–based microstructural properties.

Automated segmentation of cell nuclei was performed on hematoxylin-eosin–stained whole-slide images. The td-dMRI–derived parameters were positively correlated with pathologic measurements in a subset of 97 representative samples (cell diameter, *r* = 0.67; cellularity, *r* = 0.34; *f*_in_, *r* = 0.27; all *P* < .05) (Fig 2).

### Characteristics of td-dMRI–based Microstructural Parameters and ADC Values

**Figure 3:**
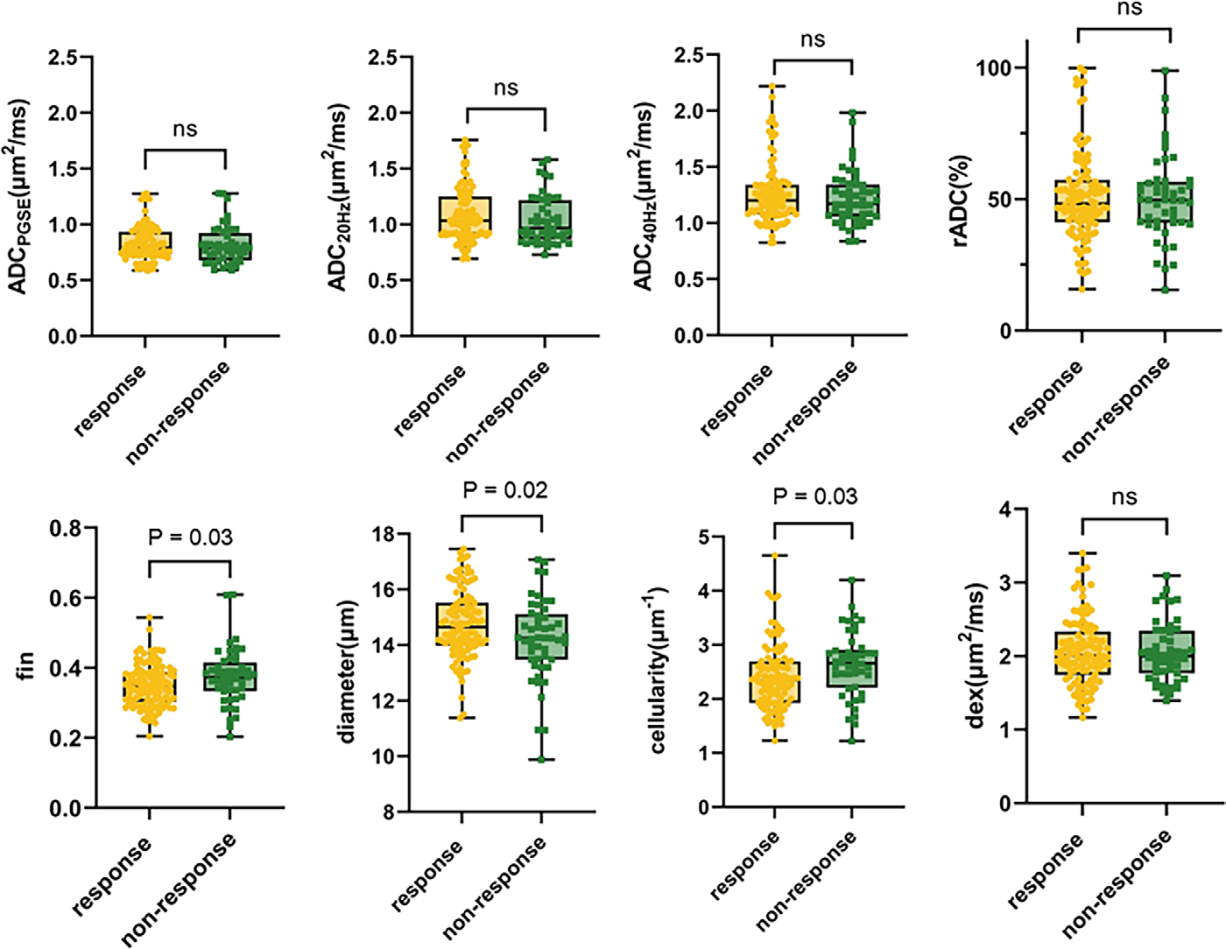
Box-and-whisker plots show comparisons of time-dependent diffusion MRI microstructural parameters between the response and nonresponse groups using Mann-Whitney *U* tests in the training set. ADC = apparent diffusion coefficient, ADC_PGSE_ = ADC value with pulsed gradient spin-echo sequence, ADC_20Hz_ = ADC value at 20 Hz, ADC_40Hz_ = ADC value at 40 Hz, dex = extracellular diffusivity, fin = intracellular fraction, ns = not significant, rADC = relative ADC change. Whiskers denote the range in each group, dots represent individual data points, boxes indicate the SD, and midlines are the median.

Within the training set, participants in the response group had larger cell diameters and lower *f*_in_ and cellularity than those in the nonresponse group (mean cell diameter: 14.65 μm ± 1.51 vs 14.25 μm ± 1.58; mean *f*_in_: 0.35 ± 0.08 vs 0.37 ± 0.07; mean cellularity: 2.34 μm^−1^ ± 0.76 vs 2.66 μm^−1^ ± 0.61; all *P* < .05). There was no evidence of a difference between the two groups for ADC_PGSE_, ADC_20Hz_, ADC_40Hz_, relative ADC change, or *D*_ex_ (response group vs nonresponse group: ADC_PGSE_ = 0.79 μm^2^/msec ± 0.20 vs 0.79 μm^2^/msec ± 0.21, *P* = .67; ADC_20Hz_ = 1.04 μm^2^/msec ± 0.34 vs 0.97 μm^2^/msec ± 0.33,* P* = .21; ADC_40Hz _ = 1.20 μm^2^/msec ± 0.26 vs 1.17 μm^2^/msec ± 0.28, *P* = .40; relative ADC change = 48.31% ± 15.71 vs 49.65% ± 15.11, *P* = .85; *D*_ex_ = 2.00 ± 0.55 vs 2.00 ± 0.56, *P* = .97) (Fig 3). This pattern was consistently observed in the internal test set (Fig S4).

**Figure 4:**
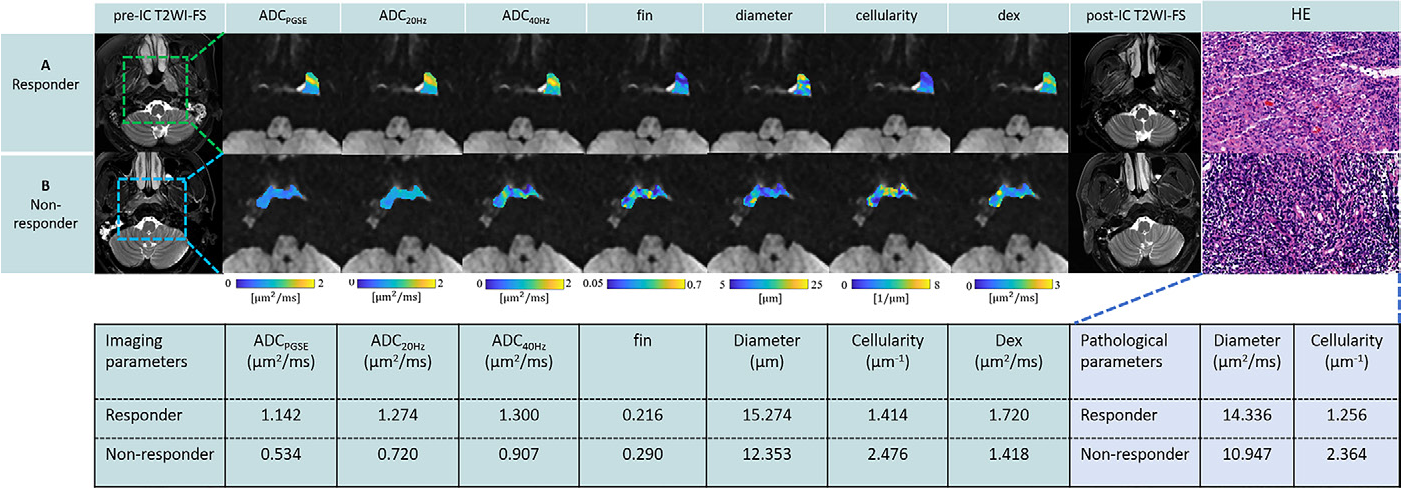
Imaging and pathologic parameters of two representative cases. **(A)** Diffusivity maps in a 54-year-old female participant with nasopharyngeal carcinoma (NPC) (T3N1M0) who underwent three cycles of chemotherapy, with follow-up MRI scan showing a partial response, placing her in the response group. **(B)** Diffusivity maps in a 41-year-old male participant with NPC (T3N1M0) who received three cycles of chemotherapy, with follow-up MRI scan showing stable disease, placing him in the nonresponse group. The magnification of the hematoxylin-eosin–stained image is ×40. ADC = apparent diffusion coefficient, ADC_PGSE_ = ADC value with the pulsed gradient spin-echo sequence, ADC_20Hz_ = ADC value at 20 Hz, ADC_40Hz_ = ADC value at 40 Hz, dex = extracellular diffusivity, fin = intracellular fraction, HE = hematoxylin-eosin, IC = induction chemotherapy, rADC = relative ADC change, T2WI-FS = T2-weighted imaging with fat suppression.

The imaging microstructural parameters with the limited spectrally edited diffusion-fitted microstructural parameter maps and ADC maps of a representative responder and nonresponder to induction chemotherapy are shown in Figure 4.

### Independent Predictors of Chemotherapy Response

In the training set, univariable logistic regression analysis revealed that the Ki-67 index (β = 0.92; OR, 2.51; 95% CI: 1.21, 4.79; *P* = .02), TSR (β = 0.80; OR, 2.22; 95% CI: 1.10, 4.45; *P* = .02), cell diameter (β = 0.21; OR, 1.15; 95% CI: 1.01, 1.45; *P* = .04), and cellularity (β = −0.37; OR, 0.63; 95% CI: 0.40, 0.99; *P* = .01) were predictors of chemotherapy response. In multivariable logistic regression incorporating variables with *P* < .05 from the univariable analysis, the TSR (β = 0.86; OR, 2.37; 95% CI: 1.17, 4.83; *P* = .02) and cellularity (β = −0.40; OR, 0.67; 95% CI: 0.47, 0.96; *P* = .03) emerged as independent predictors of a favorable response in the training set (Table 3).

**Table 3:** Univariable and Multivariable Analyses of the Associations of Clinical and Pathologic Characteristics and Time-dependent Diffusion MRI-derived Microstructural Measures with Induction Chemotherapy Response in the Training Set

Predictor	Univariable Analysis		Multivariable Analysis		
	Odds Ratio	*P* Value	β Coefficient	Odds Ratio	*P* Value
Intercept	…	…	0.37	1.44 (0.89, 2.35)	.14
Clinical and pathologic characteristics					
Age	0.99 (0.96, 1.02)	.62			
Sex					
Male	Reference				
Female	1.54 (0.72, 3.32)	.27			
Albumin level	1.23 (0.67, 2.33)	.63			
T stage					
T1	Reference				
T2	2.25 (0.80, 6.36)	.13			
T3	0.41 (0.20, 1.24)	.10			
T4	1.34 (0.66, 2.72)	.41			
N stage					
N0	Reference				
N1	1.91 (0.91, 4.02)	.10			
N2	0.69 (0.34, 1.39)	.30			
N3	0.67 (0.30, 1.47)	.32			
AJCC stage					
III	Reference				
IVa	1.02 (0.52, 2.03)	.95			
EBV DNA					
<4000	Reference				
≥4000	0.51 (0.25, 1.03)	.06			
Tumor volume	0.89 (0.45, 1.55)	.32			
Ki67					
<50%	Reference				
≥50%	2.51 (1.29, 4.79)	.02	1.82	2.41 (0.81, 7.16)	.11
TSR					
<50%	Reference				
≥50%	2.22 (1.10, 4.45)	.02	0.86	2.37 (1.17, 4.83)	.02
Chemotherapy cycles					
Two cycles	Reference				
Three cycles	0.78 (0.37, 2.75)	.28			
Time-dependent diffusion MRI microstructural parameters					
ADC_PGSE_	1.61 (0.18, 14.39)	.67			
ADC_20Hz_	2.63 (0.57, 12.15)	.21			
ADC_40Hz_	1.82 (0.46, 7.25)	.40			
rADC	1.00 (0.99, 1.02)	.55			
*f*_in_	0.44 (0.24, 1.16)	.07			
Diameter*	1.15 (1.01, 1.45)	.04			
Cellularity	0.63 (0.40, 0.99)	.01	−0.40	0.67 (0.47, 0.96)	.03
*D*_ex_	1.02 (0.47, 2.20)	.97			

Note.—Numbers in parentheses are 95% CIs. ADC = apparent diffusion coefficient, ADC_PGSE_ = ADC value with the pulsed gradient spin-echo sequence, ADC_20Hz_ = ADC value at 20 Hz, ADC_40Hz_ = ADC value at 40 Hz, AJCC = American Joint Committee on Cancer, *D_ex_* = extracellular diffusivity, *f*_in_ = intracellular fraction, rADC = relative ADC change, TSR = tumor-stroma ratio.

* Diameter was excluded from the multivariable logistic regression model because of multicollinearity.

### Model Performance

**Figure 5:**
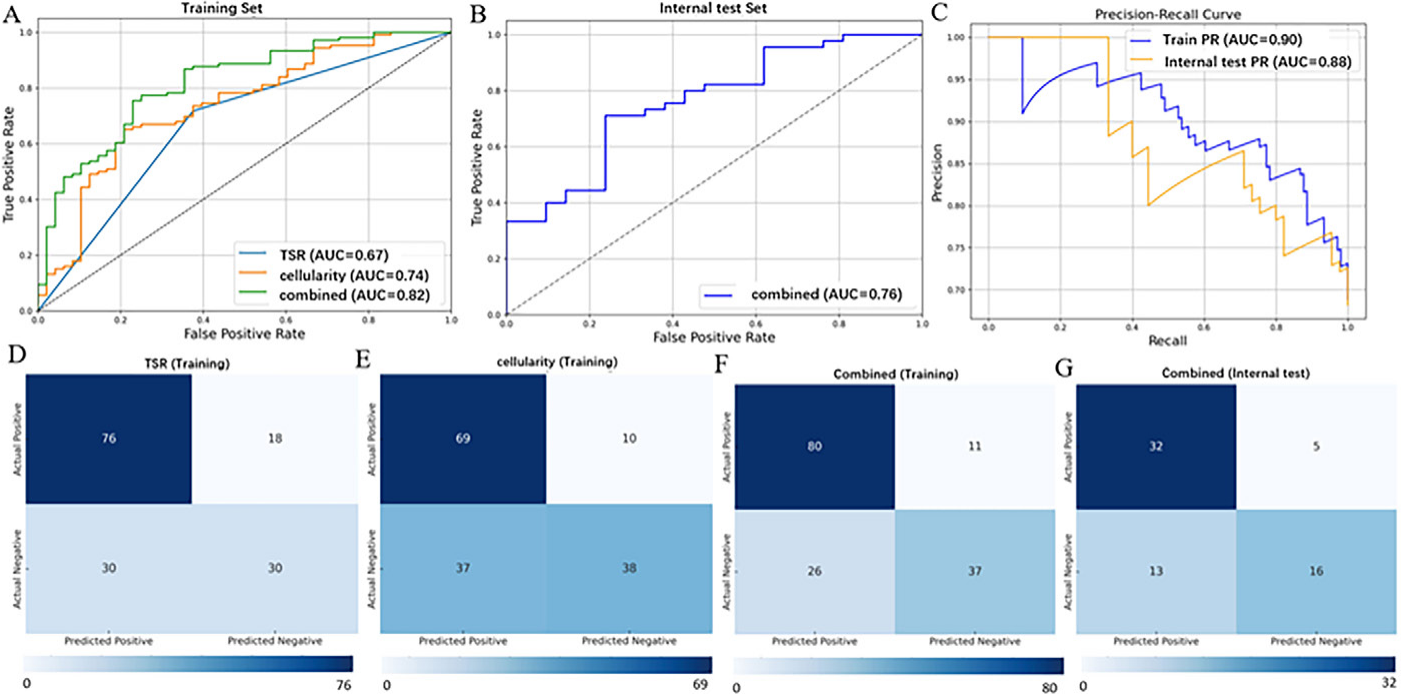
Performance of models for predicting the response to induction chemotherapy in the training and internal test sets. **(A)** Receiver operating characteristic curves of the tumor-stroma ratio (TSR), cellularity, and combined models for predicting treatment response to induction chemotherapy in the training set. **(B)** Receiver operating characteristic curve of the combined model in the internal test set. **(C)** Precision-recall (PR) curves of the combined model in both the training and internal test sets. **(D–G)** Confusion matrixes of the **(D)** TSR model in the training set, **(E)** cellularity model in the training set, **(F)** combined model in the training set, and **(G)** combined model in the internal test set. The combined model integrated both TSR and cellularity parameters. AUC = area under the receiver operating characteristic curve.

Logistic regression models were used to differentiate participants who achieved an induction chemotherapy response from those who did not. In the training set, the clinical model (TSR) achieved an AUC of 0.67 (95% CI: 0.59, 0.75), the td-dMRI–derived cellularity model yielded an AUC of 0.74 (95% CI: 0.65, 0.82), and their combination further improved the performance to an AUC of 0.82 (95% CI: 0.75, 0.89), outperforming either model alone (*P* < .05, DeLong test) (Table 4, Fig 5A). In the internal test set, the performance of the combined model (integrating both TSR and cellularity) also reached a high AUC of 0.76 (Table 4, Fig 5B).

**Table 4:** Performance of Three Models for Predicting Response to Induction Chemotherapy

Set and Model	AUC*	Sensitivity	Specificity	PPV	NPV	*P* Value^†^
Training set						
TSR	0.68 (0.59, 0.75)	76/106 (72)	30/48 (62)	76/94 (80.9)	30/60 (50.0)	<.001
Cellularity^‡^	0.74 (0.65, 0.82)	69/106 (65)	38/48 (79)	69/79 (87.3)	38/75 (50.7)	.046
Combined	0.82 (0.75, 0.89)	80/106 (75)	37/48 (77)	80/91 (87.9)	37/63 (58.7)	Reference
Internal test set Combined	0.76 (0.63, 0.87)	32/45 (71)	16/21 (76)	32/37 (86.5)	16/29 (55.2)	.15

Note.—Except where indicated, data are numbers of participants, with percentages in parentheses. The combined model integrated both TSR and cellularity parameters. AUC = area under the receiver operating characteristic curve, NPV = negative predictive value, PPV = positive predictive value, TSR = tumor-stroma ratio.

* Numbers in parentheses are 95% CIs.

^†^ *P* value indicates the significance level of the comparison of AUCs with the combined model as the reference.

^‡^ The optimal cutoff value for cellularity as determined with the Youden index was 2.44 μm^−1^.

Subgroup analyses, as shown in Figure S5, were conducted to assess whether the model’s performance remained stable across different subgroups (sex, AJCC stage, and EBV DNA). The combined model achieved high AUC values (range, 0.73–0.85) in all the subgroups (male and female; AJCC III and IVa stage; EBV DNA [EBV DNA < 4000 and EBV DNA ≥ 4000 copies/mL]) for the prediction of the response to induction chemotherapy. The precision-recall curves of the combined model in both the training and internal test sets are shown in Figure 5C. Moreover, the confusion matrix results are shown in Figure 5D–5G.

Furthermore, we evaluated the performance of the three predictive models across the entire dataset using fivefold cross-validation. The results revealed that the combined model still performed excellently, with an average AUC of 0.77 (Fig S6).

Calibration curve analysis revealed good calibration in both sets (Hosmer-Lemeshow test, all *P* > .05) (Fig S7A, S7B). Additionally, decision curve analysis suggested that the combined model provided greater clinical net benefits than the TSR model or the cellularity model alone across a threshold probability range of 0.56–0.75 in the training set and 0.36–0.75 on the internal test set (Fig S7C, S7D), with only minimal overlap among the net benefit curves in a small portion of the threshold range.

## Discussion

At present, there is no satisfactory method to accurately identify patients with locally advanced NPC who are sensitive to induction chemotherapy before treatment, although such methods are crucial for optimizing clinical management. Therefore, this study aimed to evaluate the reliability of td-dMRI–derived indicators in NPC and to investigate whether td-dMRI–derived microstructural parameters can help noninvasively predict response to induction chemotherapy. Our results showed good repeatability across measurements and high interobserver consistency, with the microstructural parameters correlated with the pathologic metrics (*r* = 0.27–0.67; *P* < .05), confirming the technical reliability of td-dMRI. Combining td-dMRI–derived cellularity with the TSR yielded a model with improved predictive performance for induction chemotherapy responders (training set AUC = 0.82), which also performed well across subgroups (AUC = 0.73–0.85) and in the internal test set (AUC = 0.76).

Owing to the inherent anatomic complexity within the nasopharynx, which predisposes it to susceptibility artifacts, and considering the potential challenges of MRI acquisition (especially oscillating gradient spin-echo sequences) in this region, we conducted a series of rigorous assessments of image quality and repeatability before quantitative analysis (16,33). Three-level validation—including qualitative image assessment, quantitative parameter consistency, and test-retest comparison—confirmed the high reliability of td-dMRI in NPC imaging. Significant correlations between td-dMRI parameters (eg, cell diameter and cellularity) and histopathology (both *P* < .05) further support its potential in future clinical research.

In our study, although the correlations between the td-dMRI parameters and the pathologic findings of cellularity and *f*_in_ were statistically significant, the strengths of these correlations were not high. This may be because the pathologic samples of NPC were obtained via endoscopic biopsy using forceps, which represent only a small portion of the tumor rather than the entire lesion. Therefore, the pathologic and imaging-derived tissue characteristics do not strictly correspond, which could contribute to the relatively weak correlations.

The TSR is a key biomarker reflecting the tumor-stroma proportion and offers a simple yet effective way to assess the effects of the tumor microenvironment on therapeutic response. In breast cancer (34) and serous ovarian cancer (19), stroma-rich tumors exhibit pronounced resistance to platinum-based chemotherapy, which is associated with poor progression-free survival and overall survival (19,34). Mechanistically, chemotherapy-resistant tumors display approximately 40% higher collagen content than their chemotherapy-sensitive counterparts (*P* < .01) (35), which may substantially impede drug penetration within tumors. These findings highlight the prognostic potential of the TSR, and combining it with td-dMRI metrics further improves treatment response prediction, supporting its use in personalized NPC therapy.

In our study, tumors with lower cellularity were more sensitive to chemotherapy, likely because of reduced proliferation and invasiveness, which is consistent with prior td-dMRI–based predictions of pathologic complete response in breast cancer (15). Other published studies have also reported that the cellularity metric can help distinguish benign and malignant tumors, with malignant lesions in prostate and breast cancers generally exhibiting higher cellular density (8,36).

Our combined model provided an AUC of 0.82 in predicting induction chemotherapy responders, which was comparable to those of a radiomics model based on conventional MRI acquisition (AUC = 0.84) (37) and a PET/CT-based model using metabolic parameters, with a C index of 0.78 (38). However, its performance is slightly lower than that of other prediction models based on conventional MRI sequences (with AUCs ranging from 0.86 to 0.95) (39–41). Nevertheless, we acknowledge that td-dMRI offers better biologic interpretability at the microstructural level. In addition to demonstrating the clinical potential of td-dMRI, these results indicate that the two incorporated variables, namely, cellularity, which reflects tumor cellular characteristics, and TSR, which represents the relative proportion of the extracellular matrix, provide valuable and complementary information for predicting treatment response, as both are essential components of the tumor microenvironment. Although the performance of the combined model based on td-dMRI and TSR was lower than that of the radiomics models derived from conventional MRI sequences, a likely explanation is that conventional radiomics models incorporate multidimensional, high-throughput features that capture complex intratumoral characteristics. In future work, we plan to extract high-dimensional features from td-dMRI parameter maps to more comprehensively reflect tumor biology, which may further enhance the predictive performance of our models.

The predictive models demonstrated good discrimination and calibration, with the combined model providing the greatest clinical net benefit across relevant risk thresholds. When the optimal cutoff was used, the models showed satisfactory sensitivity and specificity, supporting their potential to identify likely responders and nonresponders to induction chemotherapy. These findings suggest that the combined model could facilitate risk-adapted, individualized treatment decisions for NPC.

In terms of data acquisition and methodology, our study has several notable strengths. First, unlike most td-dMRI–related clinical studies with approximately 100 participants, it included a larger NPC cohort (*n* = 220), improving the reliability of the clinical conclusions. Second, beyond td-dMRI, approximately 100 pathologic analyses revealed correlations between imaging microstructural parameters with the limited spectrally edited diffusion-derived microstructural parameters and pathologic indicators, offering biologic insight into the role of imaging markers in chemotherapy sensitivity. Most importantly, despite the high hardware requirements of the td-dMRI sequence and the relative complexity of its postprocessing—which necessitates familiarity with specific algorithmic procedures—our team is actively developing a more user-friendly, graphical user interface–based postprocessing platform. As 3-T systems become increasingly prevalent in clinical practice, td-dMRI is expected to be more readily integrated into routine clinical acquisition workflows in the future.

This prospective study also has several limitations. First, as the data were acquired from a single center using one scanner, validation across different scanners and multicenter cohorts is needed. However, its relatively short acquisition time (approximately 7.5 minutes) offers potential for integration into clinical scanning protocols. Second, because NPC is primarily treated with induction chemotherapy followed by chemoradiotherapy, with surgery rarely performed, pathologic microparameters were derived from pretreatment biopsies. These localized samples may poorly correlate with imaging metrics, which reflect whole-tumor characteristics. Third, the slightly elevated false-positive rate may be due to TSR and cellularity bias, training set imbalance, feature correlation, and the combined model’s omission of key clinical, molecular, and heterogeneity factors. Fourth, the short follow-up period precluded survival analysis. Fifth, owing to the limited sample size, subgroup analyses across different induction chemotherapy regimens were not performed in our study. This aspect can be further explored in future work once a larger cohort becomes available. Similarly, the sample size of the internal validation set needs to be further expanded. Sixth, the reasonableness of the microstructural parameters’ repeatability coefficient and within‐subject coefficient of variation in our study is, to some extent, dependent on the radiologists reviewing the images and the equipment used. Future studies should reevaluate the results across different equipment and radiologists.

In summary, td-dMRI–derived microstructural parameters, particularly the combination of TSR and cellularity, effectively predicted chemotherapy response in NPC; future studies are needed to validate the findings across multiple centers and address the other limitations of the study.

## Supplemental Files

Tables S1-S5, Figures S1-S8, Appendices S1-S4

Conflicts of Interest
