# Supplementary material for: Time-dependent Diffusion MRI for Predicting Response to Induction Chemotherapy in Nasopharyngeal Carcinoma
Source: Radiol Imaging Cancer. 2026 May 8;8(3):e250579. doi: 10.1148/rycan.250579 (PMC13231216; doi:10.1148/rycan.250579)
Supplement: Tables S1-S5, Figures S1-S8, Appendices S1-S4 [file rycan250579supp.pdf]

Supplemental materials undergo peer review but are not copyedited, and may include typographical errors.

©RSNA, 2026
10.1148/rycan.250579

Table S1: Imaging Parameters for MRI Protocol

Parameters	T1WI	T2WI	T2WI-FS
Sequence	FSE	FSE	FSE
TR (ms)	600	5300	5300
TE (ms)	Min ful	85	85
FOV (cm)	22	22	22
Acquisition matrix	320*320	400*400	320*240
Slice thickness/gap	4/0.4 mm	3/0.3 mm	3/0.3 mm
Flip angle	111	111	111
bandwidth	83.33	62.5	62.5
NEX	3	3	2
Acquisition time	1:40	2:04	2:19

Supplemental materials undergo peer review but are not copyedited, and may include typographical errors.

Table S2: Time-dependent Diffusion MRI parameters for MRI Protocol

vendor	sequences	δ/△ (ms)	Cycle	f (Hz)	Effective td (ms)	b value[a]
GE	OGSE 20Hz	50/62	1	20	12.5	0 250 500 1000 1500
	OGSE 40Hz	50/62	2	40	6.25	0 250 400
	PGSE	12.66/62.1 duration/separation	/	/	58.88	0 250 500 1000 1500

Notes. OGSE = oscillating gradient spin-echo; PGSE = pulsed gradient spin-echo.

[a] Diffusion-weighted imaging data were acquired in six directions for nonzero b-values.

Supplemental materials undergo peer review but are not copyedited, and may include typographical errors.

Table S3: **Intra- and Inter-observer Agreements for Qualitative Image Quality Evaluation**

sequences	Radiolo-gists	Reading session	Qualitative image quality score					Intra-observer agreement		Inter-observer agreement	
			1	2	3	4	5	kappa value	*P* value	kappa value	*P* value
OGSE 40Hz	1	first time	2	23	30	171	27	0.98±0.02	<0.001	0.97±0.02	<0.001
		second time	3	21	34	166	29				
	2	first time	4	24	33	165	27	NA			
OGSE 20Hz	1	first time	4	26	36	162	25	0.99±0.01	<0.001	0.98±0.02	<0.001
		second time	3	25	37	161	27				
	2	first time	3	27	39	161	23	NA			
PGSE	1	first time	5	27	40	159	22	0.98±0.02	<0.001	0.99±0.01	<0.001
		second time	6	27	35	165	20				
	2	first time	5	28	37	162	21	NA			

Notes. OGSE = oscillating gradient spin-echo; PGSE = pulsed gradient spin-echo.

Supplemental materials undergo peer review but are not copyedited, and may include typographical errors.

Table S4: **Intra- and Interobserver Agreements for Microstructural Parameters and ADC Values from Time-dependent Diffusion MRI**

parameters	Radiologist	Reading session	Intra-observer agreement		Inter-observer agreement	
			ICC	*P* value	ICC	*P* value
fin	1	first time	0.92 (0.85,0.95)	<0.0001	0.78 (0.63,0.87)	<0.001
		second time				
	2	first time	/	/		
diameter	1	first time	0.91 (0.85,0.95)	<0.0001	0.85 (0.74,0.91)	<0.001
		second time				
	2	first time	/	/		
Dex	1	first time	0.92 (0.86,0.96)	<0.0001	0.81 (0.69,0.89)	<0.001
		second time				
	2	first time	/	/		
Cellularity	1	first time	0.90 (0.82,0.94)	<0.0001	0.83 (0.71,0.90)	<0.001
		second time				
	2	first time	/	/		
ADC_{PGSE}	1	first time	0.900 (0.83,0.94)	<0.0001	0.74 (0.57,0.85)	<0.001
		second time				
	2	first time	/	/		
ADC_{20Hz}	1	first time	0.88 (0.80,0.93)	<0.001	0.86 (0.75,0.92)	<0.001
		second time				
	2	first time	/	/		
ADC_{40Hz}	1	first time	0.96 (0.93,0.98)	<0.0001	0.88 (0.79,0.93)	<0.001
		second time				
	2	first time	/	/		
Relative ADC change	1	first time	0.94 (0.89,0.97)	<0.001	0.85 (0.73,0.92)	<0.001
		second time				
	2	first time	/	/		

Notes: OGSE = oscillating gradient spin-echo; PGSE = pulsed gradient spin-echo; ICC = intraclass correlation coefficient; fin = intracellular fraction; Dex = extracellular diffusivity; ADC = apparent diffusion coefficient.

Supplemental materials undergo peer review but are not copyedited, and may include typographical errors.

Table S5: Participant Characteristics in the in the Internal Test Set

Characteristics	Responders (n=45)	Nonresponders (n=21)	*P*
Age (years)	52.0 (49.0-57.0)	51.0 (38.0-56.0)	.77#
Sex, (Male, %)	32(71.1)	17(81.0)	.55*
Albumin (g/L)	45.2(39.9-56.7)	49.4(43.3-55.9)	.39#
T stage, %			.03*
T1	2(4.4)	4(19.0)	
T2	4(8.9)	0(0.0)	
T3	22(48.9)	5(23.8)	
T4	17(37.8)	12(57.1)	
N stage, %			.16*
N0	0(0)	0(0)	
N1	24(53.3)	6(28.6)	
N2	12(26.7)	8(38.1)	
N3	9(20.0)	7(33.3)	
AJCC stage, %			.43*
III	24(53.3)	9(42.9)	
IVa	21(46.7)	12(57.1)	
EBV DNA, %			.86*
<4000	29(64.4)	14(66.7)	
⩾4000	16(35.6)	7(33.3)	
Tumor volume (cm^3)	9.49(6.23-15.67)	10.24(5.87-17.54)	.65#
Ki-67 index, %			.29*
⩾50%	22(48.9)	9(42.9)	
<50%	23(51.1)	12(57.1)	
TSR, %			.06*
⩾50%	13(28.9)	11(52.4)	
<50%	32(71.1)	10(47.6)	
Chemotherapy cycle, %			.42*
Two cycles	28(62.2)	13(61.9)	
Three cycles	17(37.8)	8(37.1)	

Notes. —For categorical variables, data are numbers of participants; data in parentheses are percentages. For continuous variables, data are medians; data in parentheses are IQRs. NLR = neutrophil-to-lymphocyte ratio, PLR = platelet-to-lymphocyte ratio, SII = systemic immune inflammation index; AJCC = American Joint Committee on Cancer; EBV DNA = the copy number of Epstein–Barr virus DNA; TSR = tumor–stroma ratio; # Mann–Whitney U test; *$\chi 2$ test or Fisher exact test;

T stage and N stage were determined according to the eighth edition of the AJCC staging system for head and neck cancer.

Supplemental materials undergo peer review but are not copyedited, and may include typographical errors.

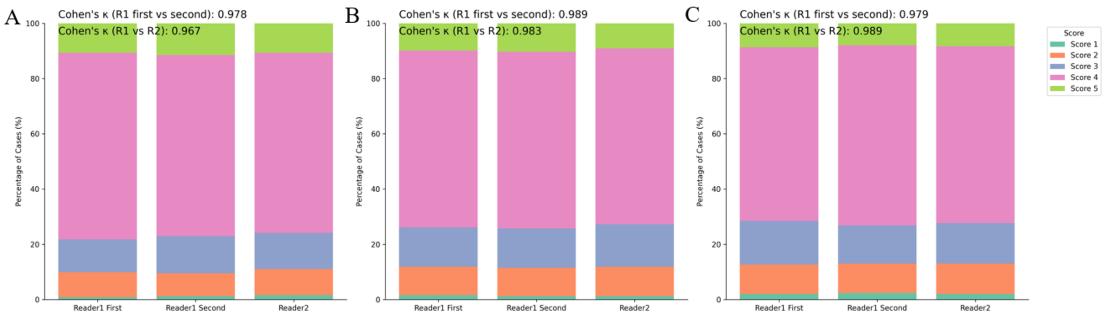

Figure S1: Stacked bar graphs display the distribution of image quality scores. Two radiologists independently rated $OGSE_{40Hz}$ (A), $OGSE_{20Hz}$ (B), and PGSE (C) using a five-point Likert scale (1 = poor, 2 = fair, 3 = moderate, 4 = good, 5 = excellent). The first radiologist performed two assessments with a 1-month interval between sessions.

Supplemental materials undergo peer review but are not copyedited, and may include typographical errors.

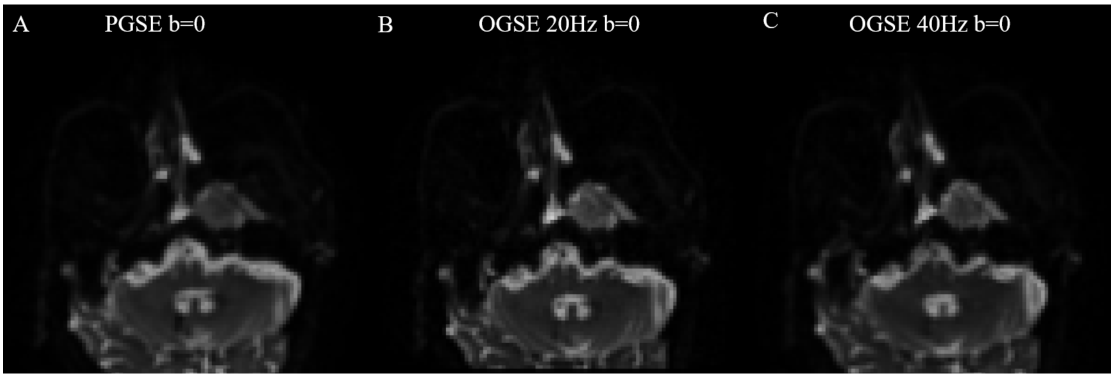

Figure S2: Td-dMRI sequence images from a patient with nasopharyngeal carcinoma.

Supplemental materials undergo peer review but are not copyedited, and may include typographical errors.

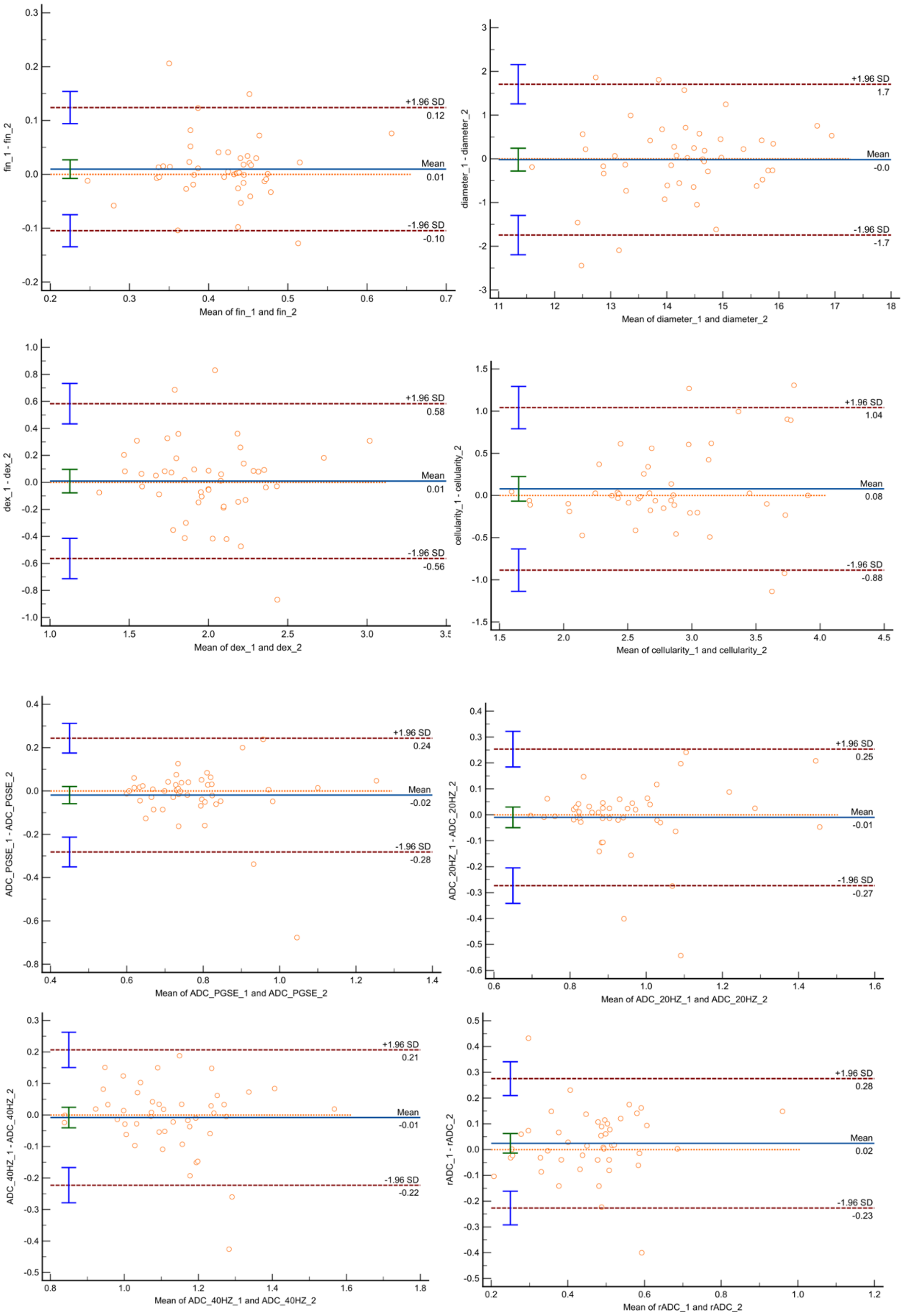

Figure S3: Bland-Altman plots of test-retest repeatability of microstructural parameters and ADC values from time-dependent diffusion MRI using IMPULSED model for Radiologist 1

Notes: fin = intracellular fraction; Dex = extracellular diffusivity; ADC = apparent diffusion

Supplemental materials undergo peer review but are not copyedited, and may include typographical errors.

coefficient; rADC = relative apparent diffusion coefficient change; The microstructural parameters and ADC values obtained from the first measurement are denoted as fin_1, diameter_1, dex_1, cellularity_1, ADC_{PGSE}_1, ADC_{20Hz}_1, ADC_{40Hz}_1, and rADC_1. Similarly, those obtained from the second measurement are denoted as fin_2, diameter_2, dex_2, cellularity_2, ADC_{PGSE}_2, ADC_{20Hz}_2, ADC_{40Hz}_2, and rADC_2.

Supplemental materials undergo peer review but are not copyedited, and may include typographical errors.

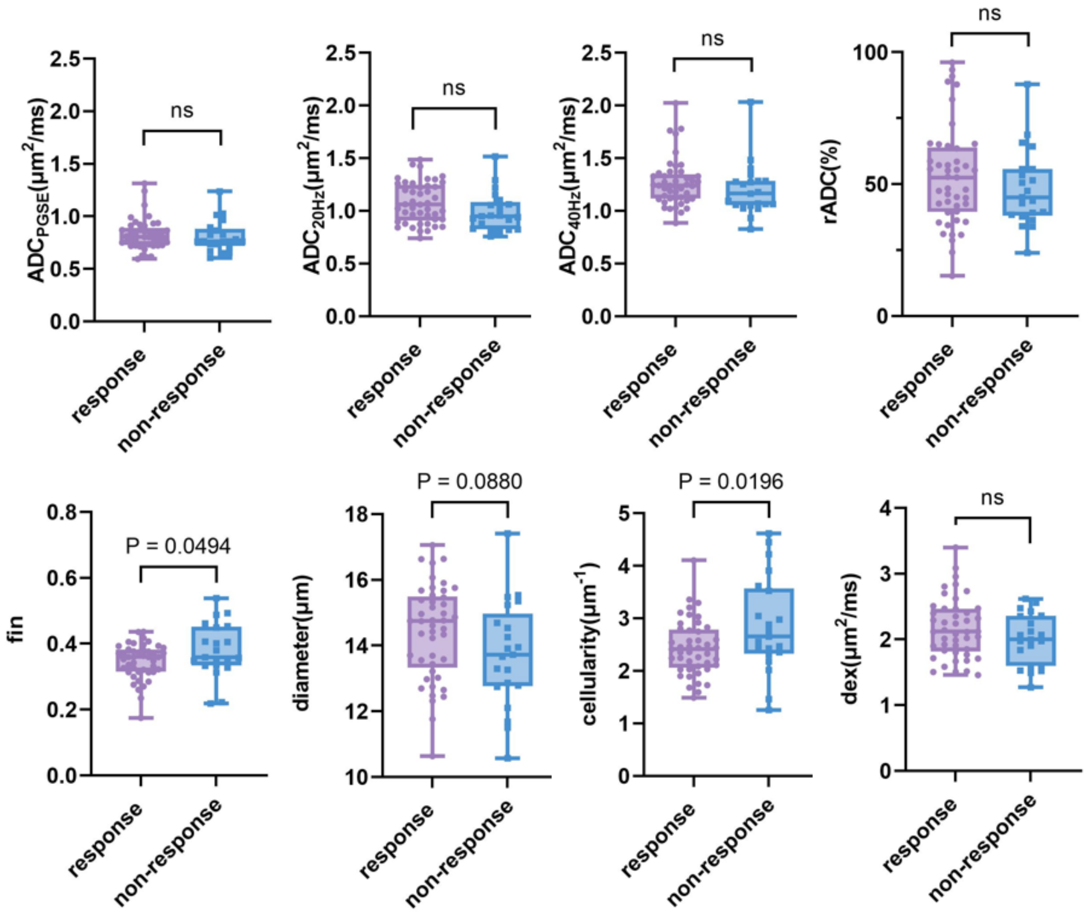

Figure S4: Box and whisker plots show comparisons of time-dependent diffusion MRI microstructural parameters between response and non-response groups using the Mann-Whitney U tests in the validation set.

Notes: ADC_{PGSE} = ADC value at pulsed gradient spin-echo; ADC_{20Hz} = ADC value at 20Hz; ADC_{40Hz} = ADC value at 40 Hz; f_{in} = intracellular fraction; D_{ex} = extracellular diffusivity; rADC = relative ADC change. Whiskers denote the range in each group, dots represent individual data points, boxes indicate the SD, and midlines are the median.

Supplemental materials undergo peer review but are not copyedited, and may include typographical errors.

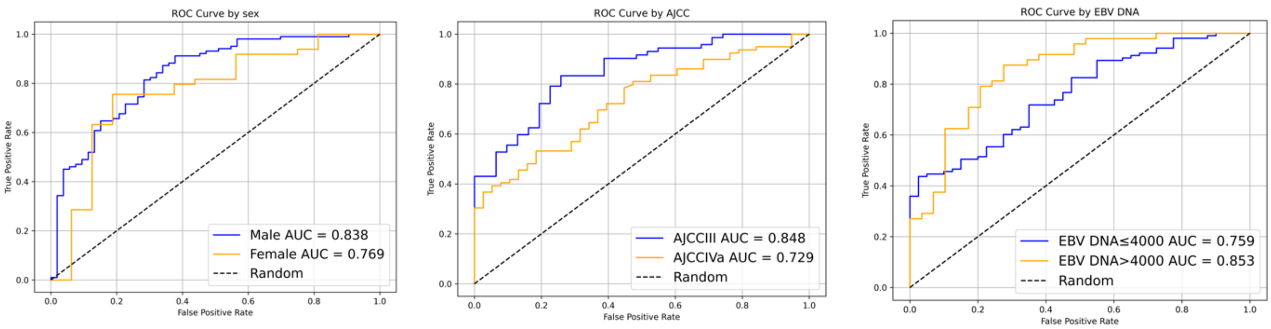

Figure S5: Receiver operating characteristic curves of the combined model in participants with different subgroups (sex, AJCC stage, EBV DNA).

Supplemental materials undergo peer review but are not copyedited, and may include typographical errors.

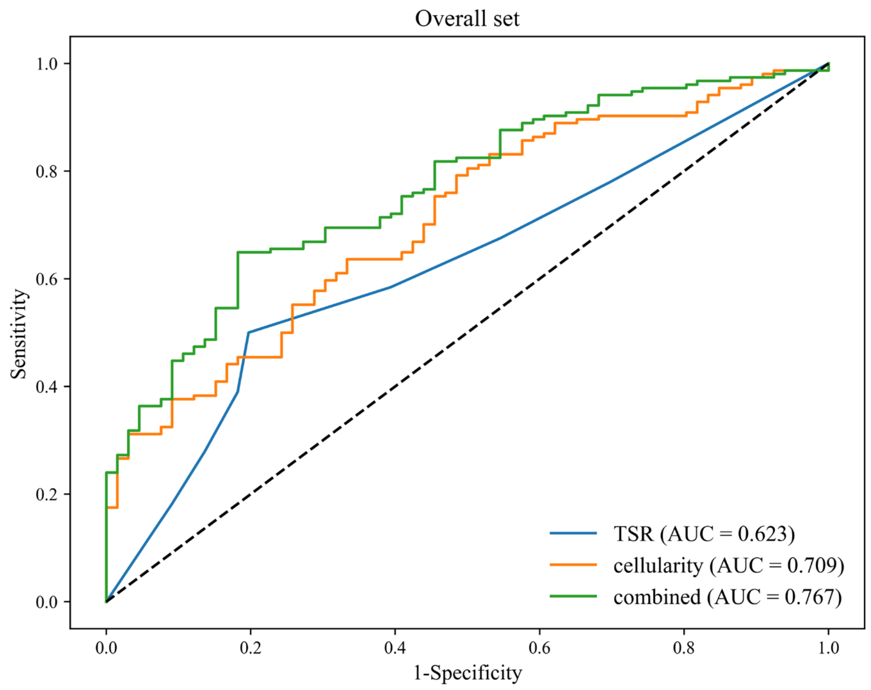

Figure S6: ROC curves of the three models predicting induction chemotherapy response in nasopharyngeal carcinoma patients, evaluated using five-fold cross-validation on the entire dataset. Notes: The combined model integrated both TSR and cellularity parameters.

Supplemental materials undergo peer review but are not copyedited, and may include typographical errors.

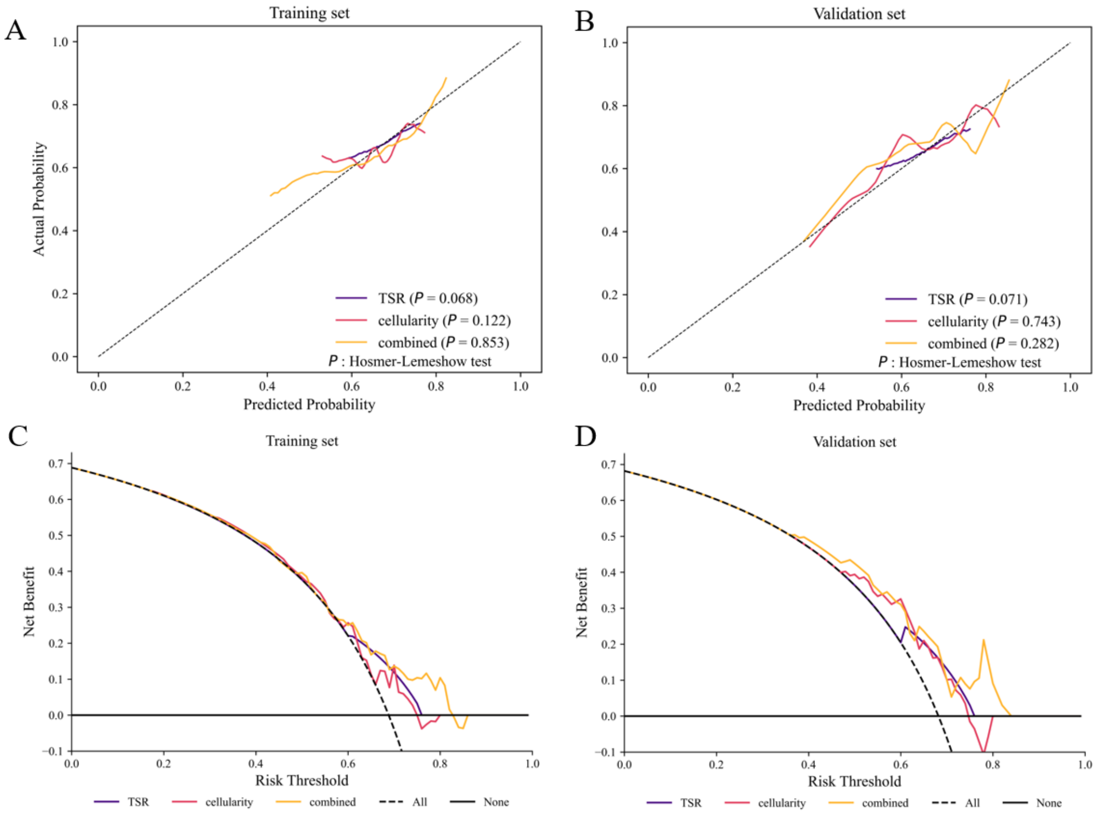

Figure S7: The calibration curves (A and B) and the decision curve analyses (C and D) for the three models (the TSR model, the cellularity model, and the combined model) predicting the response to induction chemotherapy in nasopharyngeal carcinoma at the training and validation sets.

Supplemental materials undergo peer review but are not copyedited, and may include typographical errors.

Appendix S1

TSR Assessment

Following the guidelines we first scanned the whole tumor at low magnification (×5 objective) to select an area with the highest amount of tumor-associated stroma. Then, at higher magnification using a ×10 objective we assessed an area of 3 mm^2 for the amount of tumor associated stroma so that cancer cells were present in all 4 edges of the selected microscopic field. Only a stromal area surrounded by cancer cells was assessed to ensure that tumor-associated stroma only was evaluated. In cases of a heterogenous tumor with areas of both high and low amounts of tumor-associated stroma, the stroma-high area was considered decisive for scoring the case, as recommended in the guidelines [19].

TSR was independently assessed by two pathologists (with 9 and 16 years of experience, respectively) on conventional hematoxylin and eosin-stained slides of NPC puncture samples following the established guidelines [1]. In cases of disagreement, consensus was reached through discussion. Pathologists maintained blinding to clinical/imaging data during pathological stratification of participants into stromal-rich (TSR < 50%) and stromal-poor (TSR ≥ 50%) categories [1]. This validated 50% threshold demonstrates established prognostic utility across malignancies including head and neck squamous cell carcinoma [2] and NPC [1].

References

1 Almangush A, Ruuskanen M, Hagström J et al (2024) Prognostic Significance of Tumor-associated Stroma in Nasopharyngeal Carcinoma: A Multicenter Study. Am J Surg Pathol 48:54-58.
2 Almangush A, Jouhi L, Haglund C, Hagström J, Mäkitie AA, Leivo I (2023) Tumor-stroma ratio is a promising prognostic classifier in oropharyngeal cancer. Hum Pathol 136:16-24.

Appendix S2

Estimation of the microstructural properties using IMPULSED method

a) Analytical expression of intracellular apparent restricted diffusion coefficient

Diffusion-weighted signal assuming a Gaussian phase distribution can be written as:

$$S = S_0 \exp(-\phi) \quad \text{(S1)}$$

where S_0 is the non-diffusion-weighted signal and the echo attenuation factor ϕ can be expressed as the following form based on the velocity correlation function developed by Stepisnik:

Supplemental materials undergo peer review but are not copyedited, and may include typographical errors.

$$\phi = \frac{\gamma^2}{2}\sum_k B_k \int_0^{\mathrm{TE}} dt_1 \int_0^{\mathrm{TE}} dt_2 \exp(-a_k D|t_1 - t_2|)\, g(t_1) g(t_2) \qquad \text{(S2)}$$

where γ is the gyromagnetic ratio, TE is the echo time, $g(t)$ is the time-varying diffusion gradient, D is the intrinsic diffusion coefficient, B_k and a_k are microstructure-related coefficients, which have been obtained for special shaped boundary conditions such as cylinders and spheres. Based on the Eq. (S2), the analytical expression of restricted dMRI signals under diffusion sequences with arbitrary gradient waveform can be derived, both for the sine and cosine-modulated OGSE and traditional PGSE sequences.

For the PGSE with trapezoid-shaped gradient waveforms, the analytical expression of ϕ is given as:

$$\phi = \gamma^2 G^2 \sum_k \frac{B_k}{a_k^4 D^4 t_r^2} \left\{ \begin{array}{c} 2\exp(-a_k D t_p) - 4\exp(-a_k D t_r) - 4\exp(-a_k D \Delta) \\ +2\exp(-a_k D(\Delta - t_r)) + 2\exp(-a_k D(\Delta + t_r)) \\ -\exp\left(-a_k D(\Delta - t_p)\right) - \exp\left(-a_k D(\Delta + t_p)\right) \\ -4\exp\left(-a_k D(t_r + t_p)\right) + 2\exp\left(-a_k D(2t_r + t_p)\right) \\ +2\exp\left(-a_k D(\Delta - t_r - t_p)\right) + 2\exp\left(-a_k D(\Delta + t_r + t_p)\right) \\ -\exp\left(-a_k D(\Delta - 2t_r - t_p)\right) - \exp\left(-a_k D(\Delta + 2t_r + t_p)\right) \\ -4a_k D t_r + \frac{4}{3} a_k^3 D^3 t_r^3 + 2a_k^3 D^3 t_r^2 t_p + 4 \end{array} \right\} \qquad \text{(S3)}$$

where t_r is the gradient rise time and t_p is the duration of each gradient plateau. Similarly, the analytical expression for the cosine-modulated trapezoidal OGSE sequences can also be derived from the Eq. (S2), these results have been shown previously.

In this study, tumor cells were modeled as ideal spheres, then the coefficients B_k and a_k can be expressed as:

$$B_k = \frac{2(R/\mu_k)^2}{\mu_k - 2}, \qquad a_k = \left(\frac{\mu_k}{R}\right)^2 \qquad \text{(S4)}$$

where μ_k is the kth root of $\mu J'_{3/2}(\mu) - \frac{1}{2} J_{3/2}(\mu) = 0$ and R is the tumor cell radius.

For the intracellular diffusion, the restricted dMRI signal S_r can be expressed as:

$$S_r = S_0 \exp(-\phi) = S_0 \exp(-b \cdot \mathrm{ADC_r}) \qquad \text{(S5)}$$

where b is the diffusion-weighted factor (b value) and $\mathrm{ADC_r}$ is the apparent restricted diffusion coefficient, then it can be calculated as:

$$\mathrm{ADC_r} = \frac{\phi}{b} \qquad \text{(S6)}$$

Based on Eq. (S6) and (S3), we can obtain the analytical expression of $\mathrm{ADC_r}$, which is

related to the cell diameter d or radius R, intracellular intrinsic diffusivity D_{in} ($D = D_{in}$), and specific diffusion-weighted sequences (PGSE and OGSE in this study).

b) Data analysis: IMPULSED

In this MR cytometry method, the transcytolemmal water exchange between intra- and extracellular compartments has been neglected, where the tumor cells are modeled as impermeable spheres. Then the dMRI signals are modeled as the sum of signals arising from the intra- and extracellular compartments:

$$S = S_{in} + S_{ex} \tag{S7}$$

where the water diffusion is restricted and hindered in the intra- and extracellular compartments, respectively, the corresponding signal attenuation is:

$$S_{in} = S_{in,0}\exp(-b \cdot \mathrm{ADC_r}) = f_{in}S_0\exp(-b \cdot \mathrm{ADC_r}) \tag{S8}$$

and

$$S_{ex} = S_{ex,0}\exp(-b \cdot D_{\mathrm{ex}}) = f_{ex}S_0\exp(-b \cdot D_{\mathrm{ex}}) \tag{S9}$$

where $S_{in,0}$ and $S_{ex,0}$ are non-diffusion-weighted signals, f_{in} and f_{ex} are the volume fractions of intra- and extracellular compartments, and D_{ex} is the extracellular hindered diffusivity. Note that $f_{in} + f_{ex} = 1$, the overall signal S can be expressed as:

$$S = S_0(f_{in} \cdot \exp(-b \cdot \mathrm{ADC_r}) + (1 - f_{in}) \cdot \exp(-b \cdot D_{\mathrm{ex}})) \tag{S10}$$

Figure S8 provides a schematic diagram of the IMPULSED biophysical modeling and the corresponding acquisition sequence.

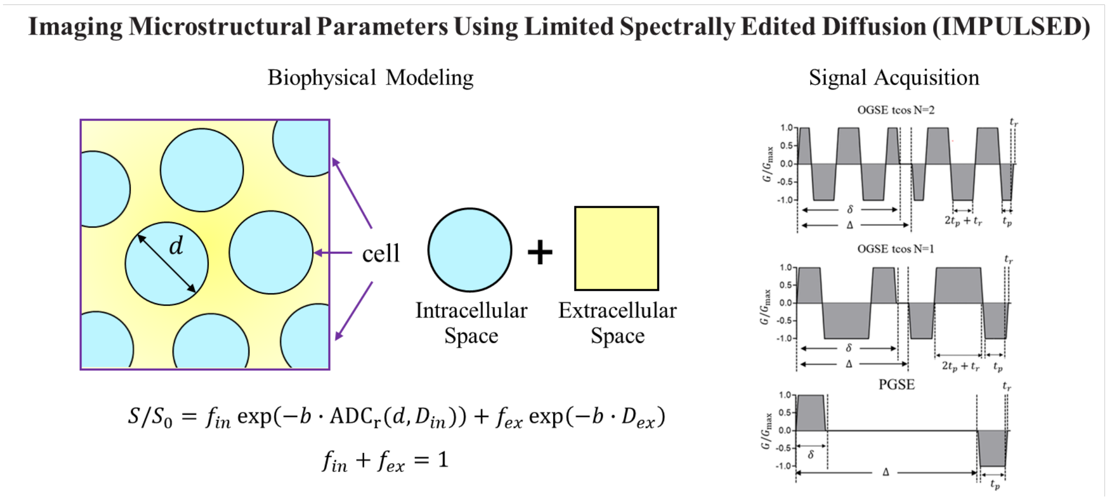

Figure S8. Schematic diagram of biophysical modeling and signal acquisition in IMPULSED

Appendix S3

Supplemental materials undergo peer review but are not copyedited, and may include typographical errors.

Treatment regimen

The induction chemotherapy regimens for locally advanced NPC were as follows: 59 participants were administered TPC, comprising paclitaxel (135-175 mg/m^2) on day 1, cisplatin (80 mg/m^2) on day 1, and capecitabine (1000 mg/m^2) twice daily from days 1 to 14; 75 participants received TP, including paclitaxel (135-175 mg/m^2) on day 1 and cisplatin (80 mg/m^2) on day 1; 71 participants were treated with GP, which involved gemcitabine (1000 mg/m^2) on days 1 and 8, and cisplatin (80 mg/m^2) on day 1; and 15 participants received DPF, which included docetaxel (75 mg/m^2) and cisplatin (80 mg/m^2) on day 1, and fluorouracil (750 mg/m^2) on days 1 to 5.

Assessment of Tumor Response

Complete response was defined as disappearance of the target lesion in the nasopharyngeal region. Partial response was defined as a decrease of at least 30% in the diameter of the target lesion by comparing with the baseline diameter of pretreatment. Progressive disease was defined as an increase of at least 20% in the diameter of target lesion or the appearance of one or more new lesions. Stable disease was defined as neither sufficient shrinkage to qualify for partial response nor sufficient increase to qualify for progressive disease.

Appendix S4

Supplementary Methods: Pretraining of the Nuclei Segmentation Model [R-S1]

The nuclei segmentation model used in this study was developed based on a conditional generative adversarial network framework following the pix2pix image-to-image translation paradigm. This framework is well suited for paired image–mask learning tasks and enables simultaneous enforcement of global structural consistency and local boundary fidelity, which is particularly advantageous for accurately segmenting densely distributed and morphologically heterogeneous cell nuclei.

The generator was implemented using a U-Net architecture with skip connections. The encoder extracts multi-scale hierarchical features, while the decoder progressively reconstructs high-resolution segmentation masks by integrating contextual information with fine spatial details transmitted through the skip connections. The discriminator

Supplemental materials undergo peer review but are not copyedited, and may include typographical errors.

adopted a PatchGAN architecture, which evaluates the realism of local image patches, thereby enhancing local texture consistency and sharpening nuclear boundaries during training.

Pretraining was conducted on the publicly available CoNic Challenge 2022 dataset, which provides paired hematoxylin and eosin (H&E)–stained histopathology images and pixel-level expert annotations of cell nuclei. The dataset comprises samples from multiple institutions and exhibits substantial variability in staining protocols, tissue types, and nuclear morphology. This diversity provides a robust foundation for learning generalizable nuclear representations and reduces dependence on dataset-specific appearance characteristics.

Model training was performed on a workstation equipped with six NVIDIA GeForce RTX 3090 GPUs. The implementation was based on PyTorch 2.3, Python 3.11, and CUDA 12.5. The network was trained for 200 epochs using the Adam optimizer with an initial learning rate of 2×10^{-4}. The exponential decay rates for the first- and second-order moment estimates were set to $\beta_1 = 0.5$ and $\beta_2 = 0.999$, respectively. The training objective consisted of a weighted combination of adversarial loss and L1 loss, with the L1 loss weight λ set to 100. The batch size was fixed at 2.

After pretraining, the model achieved a pixel accuracy of 0.9759 and a Dice coefficient of 0.8838 on the training set, and 0.9091 and 0.7191, respectively, on the validation set, indicating satisfactory baseline performance and generalization capability. Pretraining on the CoNic dataset effectively facilitated knowledge transfer to private datasets, particularly in scenarios where pixel-level annotations are limited and domain shifts are present.

During inference, owing to the substantially larger image size compared with the public dataset, an overlapping sliding-window strategy was employed. Large images were decomposed into partially overlapping patches, each independently processed by the pretrained generator and subsequently merged. This strategy effectively mitigates boundary artifacts between adjacent patches and ensures spatial continuity and global consistency in the final segmentation results.

Supplemental materials undergo peer review but are not copyedited, and may include typographical errors.

[R-S1] Isola P, Zhu J Y, Zhou T, et al. Image-to-image translation with conditional adversarial networks[C]//Proceedings of the IEEE conference on computer vision and pattern recognition. 2017: 1125-1134.s
